# The Impact of Dietary Melatonin on Heart and Lung Telomere Length and Shelterin Protein Gene Expression of Pulmonary Hypertensive Broiler Chickens

**DOI:** 10.1002/vms3.70355

**Published:** 2025-04-21

**Authors:** Peyman Gheytaspour, Shahab Bahadoran, Hossein Hassanpour

**Affiliations:** ^1^ Department of Clinical Sciences Faculty of Veterinary Medicine Shahrekord University Shahrekord Iran; ^2^ Department of Basic Sciences Faculty of Veterinary Medicine Shahrekord University Shahrekord Iran; ^3^ Health Equity Research Center Shahed University Tehran Iran

**Keywords:** ascites, congestive heart failure, Melatonin, shelterin complex, telomere, telosome

## Abstract

**Objectives:**

Pulmonary hypertension syndrome (PHS) is a common metabolic disease in broiler chickens linked to oxidative stress. This study explored the potential of melatonin, an antioxidant, to improve PHS response and telomere structure in chickens with cold‐induced PHS.

**Methods:**

We investigated the effects of dietary melatonin supplementation on telomere length and the expression of genes related to telomere protection (shelterin genes) in the heart and lungs of broiler chickens with PHS.

**Results:**

Melatonin supplementation improved telomere length in the heart tissue of chickens with PHS. We also observed changes in the expression of genes (TRF1, RAP1, and TPP1) responsible for protecting telomeres, suggesting a potential mechanism for melatonin's beneficial effects. Melatonin's impact was more pronounced in the heart than in the lungs.

**Conclusions:**

Melatonin may help protect cardiac cells during PHS by improving telomere length and influencing the activity of genes involved in telomere protection. These findings suggest that melatonin could be a valuable tool in managing heart cell dysfunction associated with PHS in poultry.

## Graphical abstract

Melatonin supplementation improves telomere length and shelterin gene expression in the heart of broiler chickens with pulmonary hypertension, suggesting its potential to mitigate cardiac dysfunction and reduce PHS severity.

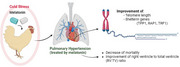

## 1 Introduction

Pulmonary hypertension syndrome (PHS), also known as ascites, is a significant health issue in the poultry industry, particularly in broiler chickens. The intensive selection for traits such as rapid growth and high feed conversion efficiency in broilers has increased their susceptibility to metabolic disorders, including ascites. The rapid growth rate leads to a high basal metabolic rate, thereby increasing the oxygen demand of tissues (Hassanzadeh et al. 2014). This higher demand necessitates an increased cardiac output, putting substantial overload on the cardiovascular system. As the heart intensifies its activity to accommodate the increased oxygen demand, the pressure within the pulmonary arteries elevates and leads to pulmonary hypertension. The increased pulmonary pressure imposes an overload on the right ventricle, resulting in right ventricular hypertrophy (Ezzulddin 2023). However, this compensatory hypertrophy can eventually lead to right ventricular failure. As the right ventricle of the heart fails to effectively pump blood, the pressure within the venous system rises, leading to venous congestion. The increased hydrostatic pressure within the capillaries causes fluid to leak from the blood vessels into the surrounding tissues and body cavities (Mahjoor and Hadipoor 2020; Miao et al. 2022).

Telomeres are unique structures found at the ends of chromosomes, composed of repetitive TTAGGG sequences. They serve essential functions in safeguarding chromosomal integrity by preventing enzymatic degradation of chromosome ends and maintaining genomic stability (Badmus et al. 2021). Integral to this protective role is the presence of telomere‐binding proteins, known as the shelterin complex. The shelterin complex comprises several proteins, including telomere repeat binding factors TRF1 and TRF2, repressor/activator protein RAP1, TRF‐interacting nuclear protein TIN2, telomere protection protein TPP1, and protection of telomeres protein POT1. These proteins collaborate to maintain the structural and functional integrity of telomeres, thereby preventing cellular abnormalities. Expression levels of shelterin components profoundly influence telomere length, thereby impacting cellular function and longevity. Thus, the shelterin complex would be a critical regulator of telomere dynamics and cellular homeostasis (Mir et al. 2020). Telomere shortening and shelterin dysfunction contribute to endothelial dysfunction, senescence, oxidative stress, and inflammation, all of which are key factors in PHS and cardiovascular pathology (Gruber et al. 2021; Hassanpour et al. 2015; 2023; Tarazón et al. 2021).

Melatonin (N‐acetyl‐5‐methoxytryptamine) is an indole amine derivative produced by the pineal gland. Interest in melatonin as a potential therapeutic agent has grown due to its diverse physiological effects. It plays a crucial role in regulating several physiological functions such as circadian rhythms, immune responses, oxidative stress, apoptosis, and mitochondrial function. Melatonin is capable of entering cellular organelles and acts as a scavenger of reactive oxygen species (ROS) (Calislar et al. 2018; Pohanka 2022). Melatonin may protect telomeres in cardiac cells by reducing oxidative damage and inflammation and improving shelterin protein gene expression thereby mitigating the effects of PHS in broiler chickens. This study induced PHS in broiler chickens using cold stress. Then, it assessed the impact of dietary melatonin on relative telomere size and transcript of shelterin genes (TRF1, TRF2, RAP1, POT1, and TPP1) in the lungs and heart of these chickens. Despite the known antioxidant benefits of melatonin, the specific mechanisms by which it impacts telomere health and shelterin gene expression in PHS‐affected broiler chickens remain unclear. Addressing this knowledge gap is crucial, as it could pave the way for the development of targeted melatonin supplementation strategies to improve cardiac health and reduce the incidence of PHS in poultry.

## 2 Materials and Methods

### 2.1 Bird Management and Induction of PHS by Cold Stress

A total of 108 one‐day‐old broiler chicks of the Ross 308 strain were randomly allocated into three groups (three pens/group): a control group and two treatment groups, each consisting of 36 birds (12 birds/pen). This sample size was determined to be sufficient to detect meaningful differences between treatment groups, considering the expected variability in PHS development and the magnitude of effects observed in prior research on melatonin supplementation in poultry (Akbarian et al. 2014; Patil et al. 2013). The treatment groups received diets supplemented with melatonin powder (≥98% purity, confirmed by thin‐layer chromatography, provided by RazakPharma Co., Tehran, Iran) at the concentrations of 20 and 40 mg/kg, corresponding to control, melatonin‐0.2% and melatonin‐0.4% groups, based on previous studies (Mohit et al. 2014; Patil et al. 2013). These concentrations have been shown to be well tolerated by broiler chickens, making them suitable for assessing the therapeutic potential of melatonin. All groups were housed in a single room under uniform conditions with a standard ration provided (Table 1). Cold stress, designed to induce PHS, was applied by gradually lowering the temperature over the 42‐day rearing period, following the methodology of Hassanpour et al. (2023). The temperature was systematically decreased by ∼17°C over the first 21 days (from 32 to 15°C) and then maintained at 15°C until the conclusion of the experiment. Hassanpour et al. (2023) validated this method as effective in reliably inducing PHS symptoms. They reported that lower temperatures increase metabolic demands, leading to cardiac workload, right ventricular hypertrophy, and dilation which are key factors in the development of PHS.

**TABLE 1:** The composition of basal diet fed to the broiler chickens during the entire experiment.

Ingredient	1–10	11–28	29–42
Corn	47.16	49.63	55.62
Soybean meal (42% CP)	42.99	38.9	33.47
Soybean oil	5.36	7.33	6.99
DL‐methionine	0.34	0.3	0.22
Lysine HCL	0.08	0.1	0.1
Vitamin premix^a^	0.3	0.3	0.3
Mineral premix^b^	0.3	0.3	0.3
Common salt	0.33	0.34	0.34
Oyster shell	1.17	1.07	1.05
Dicalcium phosphate	1.97	1.73	1.61
Total	100	100	100
Nutrient composition			
AME (Kcal/kg)	3010	3175	3225
Crude protein (%)	22.5	21	19.2
Ca (%)	1	0.9	0.85
Available phosphorus	0.5	0.45	0.42
Na (%)	0.16	0.16	0.16
Arg (%)	1.64	1.52	1.37
Lys (%)	1.45	1.35	1.21
Met (%)	0.70	0.64	0.54
Met + Cys (%)	1.07	0.99	0.86
Threonine (%)	0.96	0.89	0.82

^a^Supplied per kg of diet: vitamin A (trans‐retinyl acetate), 12,000 IU; vitamin D3 (cholcalciferol), 2000 IU; vitamin E (DL‐α‐tocopherol), 60 IU; vitamin K3 (menadione nicotinamide bisulphite), 3 mg; thiamine (thiamine‐mononitrate), 2.5 mg; riboflavin, 6.5.0 mg; pantothenic acid (D‐Ca pantothenate), 10 mg; niacin (nicotinic acid), 30 mg; pyridoxine (pyridoxine. HCl), 3 mg; folic acid, 2.0 mg; biotin, 0.2 mg; vitamin B12 (cyanocobalamin), 0.016 mg; choline (choline chloride 60%), 400 mg.

^b^ Supplied per kg of diet: Zn (zinc sulphate), 85 mg; Mn (manganese oxide), 100 mg; Cu (copper sulphate), 10 mg; Fe (iron sulphate), 50 mg; I (calcium iodate), 1 mg; Se (sodium selenite), 0.3 mg.

Throughout the study, total mortality was recorded, and necropsies were performed on deceased birds to assess lesions indicative of PHS.

### 2.2 Tissue Sampling and Estimation of the PHS Index

At 42 days, 12 birds from each experimental group (four birds per pen) were randomly selected and euthanized. Lung and heart tissues were dissected under sterile conditions. The heart ventricles were harvested according to Hassanpour et al. (2009), and the right ventricular to total ventricular weight ratio (RV:TV ratio) was determined. This ratio is a key indicator of the severity of PHS. An RV:TV ratio between 0.25 and 0.28 signifies developmental pulmonary hypertension in chickens, whereas a ratio of 0.29 or higher indicates clinical PHS, including clinical and necropsy signs of ascites. The lung and right ventricular tissues were then rapidly frozen in liquid nitrogen and stored at −70°C until RNA extraction.

### 2.3 DNA Extraction and Quantitative Real‐Time Polymerase Chain Reaction

DNA was extracted from lung and right ventricular heart tissues using the High Yield DNA Purification Kit (DNP Kit; SinaClon BioScience, Karaj, Iran) according to the manufacturer's protocol. Following extraction, the DNA pellet was resuspended in 50 µL of solvent buffer and subsequently preserved at −70°C pending PCR analysis.

Relative telomere length was measured using qPCR with a SYBR Green Real‐Time PCR kit (Parstous Co., Mashhad, Iran). Table 2 displays the characteristics of primers for telomere and the reference gene tyrosine 3‐monooxygenase/tryptophan 5‐monooxygenase activation protein zeta (YWHAZ) which were synthesized according to Hassanpour et al. (2023). Each PCR amplification was performed in a final volume of 10 µL, with triplicate reactions for both telomere and YWHAZ per sample, using a Rotor‐Gene 6000 thermocycler (Qiagen, Australia). The final primer concentrations and the PCR conditions were as specified by Hassanpour et al. (2023). A no‐template control was included to ensure the absence of contamination. Primer specificity was confirmed through melt curve analysis, which exhibited distinct peaks for both telomere and YWHAZ (Figure S1). Relative telomere length was calculated by determining the T/S ratio for each sample, following the method described by Cawthon (2002).

**TABLE 2:** Primers used for quantitative real‐time PCR analysis of chicken telomere and mRNAs.

Target	Primers	Tm (oC)	GC (%)	Ta (oC)	PCR product (bp)	PCR efficiency (%)	Accession No.
Telomere	5′‐CGGTTTGTTTGGGTTTGGGTTTG GGTTTGGGTTTGGGTT‐3′	67.6	48.7	54	78	98.2	—
	5′‐GGCTTGCCTTACCCTTACCCTTA CCCTTACCCTTACCCT‐3′	69.7	53.8				
POT1	5′‐CCTACCCGCTAACAAAACTTGG‐3′	59.5	50.0	61	160	99.1	NM_206992.1
	5′‐GCAACTATGAAGGTGTCCCCA‐3′	60.0	52.3				
RAP1	5′‐GTGGTACGGCGCTATGGAAG‐3′	60.8	60.0	56	194	99.6	NM_001397274.1
	5′‐GTCACTTCCTGACTCGGAGC‐3′	60.1	60.0				
TPP1	5′‐CTCTTCGCTTCGGGTGATGA‐3′	59.8	55.0	56	157	98.5	XM_040657790.2
	5′‐AATCCGTCACTTCTGCCGTC‐3′	60.3	55.0				
TRF1	5′‐CGAAGAAGACAGCCGTGGAC‐3′	61.0	60.0	62	130	99.8	NM_204380.3
	5′‐TCAACATGACGCTGGTTCGG‐3′	60.9	55.0				
TRF2	5′‐AGGGATCTGGAAAGGACGC‐3′	59.1	57.8	56	183	98.7	NM_001271892.2
	5′‐CCGTCTCTTGGTTCTGTGGG‐3′	60.3	60.0				
YWHAZ	5′‐AGGAGCCGAGCTGTCCAATG‐3′	62.2	60.0	61	83	99.3	NM_001031343.1
	5′‐TCCAAGATGACCTACGGGCTC‐3′	61.3	57.1				

Abbreviations: POT1, protection of telomeres protein 1; RAP1, repressor/activator protein 1; TIN2, TRF‐interacting nuclear protein; TPP1, telomere protection protein 1; TRF, telomere repeat binding factor.

### 2.4 RNA Extraction, cDNA Synthesis, and RT‐qPCR

To extract total RNA, the lung and heart tissues were homogenized and digested with RNAxPLUS solution (Sinaclon Bioscience), followed by adding chloroform and then centrifuged. The supernatant was mixed with isopropanol to precipitate the RNA following centrifugation. The resulting RNA pellet was washed with 75% ethanol, centrifuged again, and dissolved in distilled water. RNA quantity was determined by spectrophotometry at an absorbance ratio (A260/280) of 1.8–2.0, indicating suitability for cDNA synthesis. The extracted RNA was immediately reverse‐transcribed using the Easy cDNA Reverse Transcription Kit (Parstous Co.) (Ahmadipour et al. 2021).

The transcripts for TRF1, TRF2, RAP1, POT1, TPP1, and YWHAZ (used as an internal control) were amplified via Real time‐quantitative polymerase chain reaction (RT‐qPCR) utilizing the SYBR Green Real‐Time PCR kit (Parstous Co.). The specific primers (Table 2) and thermal cycling conditions were implemented according to Hassanpour et al. (2023). Amplification of the cDNA samples was conducted in triplicate using a real‐time PCR cycler (Rotor‐Gene Q 6000; Qiagen, USA). To ensure the integrity of the PCR reagents, no‐template controls, and no‐reverse transcriptase controls were included to detect any potential contamination. The melt curves of shelterin and internal control (YWHAZ) genes following amplification showed a single peak (Figure S1). The threshold cycle number (Ct) was determined, and the efficiency of the PCR reactions was calculated using LinRegPCR software version 2012.0 (Amsterdam, Netherlands). Relative transcript levels (target gene/YWHAZ) were quantified using the efficiency‐adjusted Pfaffl method.

### 2.5 Statistical Analysis

All data points are expressed as means ± standard error (SE). Statistical analyses were performed using SPSS software (version 26.0; IBM Corp.). To check the normality of data, the Kolmogorov–Smirnov test was performed. The significance of differences between the means of the experimental groups was assessed by a one‐way analysis of variance (ANOVA). The correlation between various parameters and the RV:TV ratio was analyzed using linear regression. The principle component analysis (PCA) was used to find the shelterin protein genes that most effectively contributed to variance in pulmonary hypertension severity among broilers. Two principle components (PC1 and PC2) were found from five shelterin components. To determine which components were most significant, PC1 was calculated to intergroup variation in pulmonary hypertensive chickens following dietary supplementation by melatonin. PCA was plotted by https://www.bioinformatics.com.cn/en. Differences were considered statistically significant at *p* < 0.05. The effect sizes were calculated and visualized as forest plots using Stata software version 11.2 (Stata Corp., College Station, TX, USA).

## 3 Results

The study revealed that dietary melatonin supplementation at a concentration of 0.4% had a notable impact on broiler chickens with induced PHS.

### 3.1 Index of PHS, Morbidity, and Mortality Rates

At 42 days of age, chickens in the melatonin‐0.4% group exhibited a significantly decreased right RV:TV ratio compared to the control group (*p* = 0.004), indicating a reduction in PHS severity. While the 0.2% melatonin group also showed a decrease, it was not statistically significant (*p* = 0.056) (Table 3). The morbidity rates of developmental PHS (defined as an RV:TV ratio ranging from 0.25 to 0.28) and clinical PHS (characterized by an RV:TV ratio greater than 0.28) in experimental groups along with the ascites (Figure S2) mortality rates are presented in Table 3.

**TABLE 3:** Mortality rate and right ventricle to total ventricles (RV:TV) ratio in the experimental groups of broiler chickens at 42 days.

Group	Mortality	RV:TV ratio			
		<0.25	≥0.25 < 0.29	≥0.29	Mean ± SE
PHS	12 (33.3%)	6/21 (28.6%)	5/21 (23.8%)	10 /21 (47.6%)	0.301 ± 0.014a
Melatonin‐0.2%	8 (22.2%)	10/21 (47.6%)	5/21 (23.8%)	6/21 (28.5%)	0.270 ± 0.012ab
Melatonin‐0.4%	5 (13.9%)	13/21 (61.9%)	4/21 (19.1%)	4/21 (19.1%)	0.252 ± 0.008b

*Note*: Lowercase letters indicate a significant difference between treatments (p < 0.05). The RV:TV ratio significantly decreased in the melatonin‐0.4% group compared to the control group.

Abbreviation: PHS, pulmonary hypertension syndrome.

### 3.2 Estimation of Relative Telomere Length and Expression of Shelterin Genes

The real‐time PCR results for the POT1, TRF1, TRF2, RAP1, and TPP1 genes, along with telomere length, are detailed in Table 4. The most prominent effects of melatonin were observed in the heart tissue. Specifically, the melatonin‐0.4% treatment group showed significantly increased relative telomere length and elevated transcript levels of the TRF1, RAP1, and TPP1 genes in right ventricular tissue compared to both the control and melatonin‐0.2% groups (*p* < 0.05). This suggests a protective effect of melatonin on cardiac cells at the higher dosage. In contrast, expression levels of POT1 and TRF2 did not significantly differ between the groups (*p* > 0.05).

**TABLE 4:** Relative telomere length and gene expression (/YWHAZ) in the experimental groups.

Target	*n*	PHS	Melatonin‐0.2%	Melatonin‐0.4%	*p* value
**Heart**					
Telomere	12	0.367 ± 0.016a	0.376 ± 0.047a	0.435 ± 0.024b	0.031
POT1	12	0.104 ± 0.025	0.138 ± 0.020	0.077 ± 0.007	0.530
RAP1	12	0.67 ± 0.07a	1.57 ± 0.67a	3.41 ± 0.62b	0.003
TPP1	12	0.063 ± 0.005a	0.085 ± 0.013a	0.168 ± 0.015b	0.001
TRF1	12	0.132 ± 0.028a	0.204 ± 0.075a	0.416 ± 0.121b	0.049
TRF2	12	1.62 ± 0.691	1.11± 0.163	1.55 ± 0.252	0.656
**Lung**					
Telomere	12	0.326 ± 0.021	0.296 ± 0.019	0.324 ± 0.027	0.593
POT1	12	3.21 ± 1.09	2.62 ± 0.71	3.45 ± 1.36	0.861
RAP1	12	0.677 ± 0.124	0.669 ± 0.132	0.698 ± 0.223	0.992
TPP1	12	0.046 ± 0.011	0.053 ± 0.034	0.056 ± 0.010	0.961
TRF1	12	1.34 ± 1.07	1.20 ± 0.59	1.61 ± 1.20	0.956
TRF2	12	9.93 ± 4.23	8.41 ± 2.42	9.42 ± 2.71	0.943

*Note*: Values are means ± SE; *n*, number of chickens. Lowercase letters indicate a significant difference between treatments (p < 0.05). At 42 days, the melatonin‐0.4% group only exhibited significant elevations of telomere length and expression of TRF1, RAP1, and TPP1 genes in the right ventricular tissue, compared to the control and melatonin‐0.2% groups.

Abbreviation: PHS, pulmonary hypertension syndrome.

It is important to note that unlike the heart, no significant changes were observed in relative telomere length or transcript levels of POT1, TRF1, TRF2, RAP1, and TPP1 genes in the lung tissue across the experimental groups (*p* > 0.05).

**FIGURE 1:**
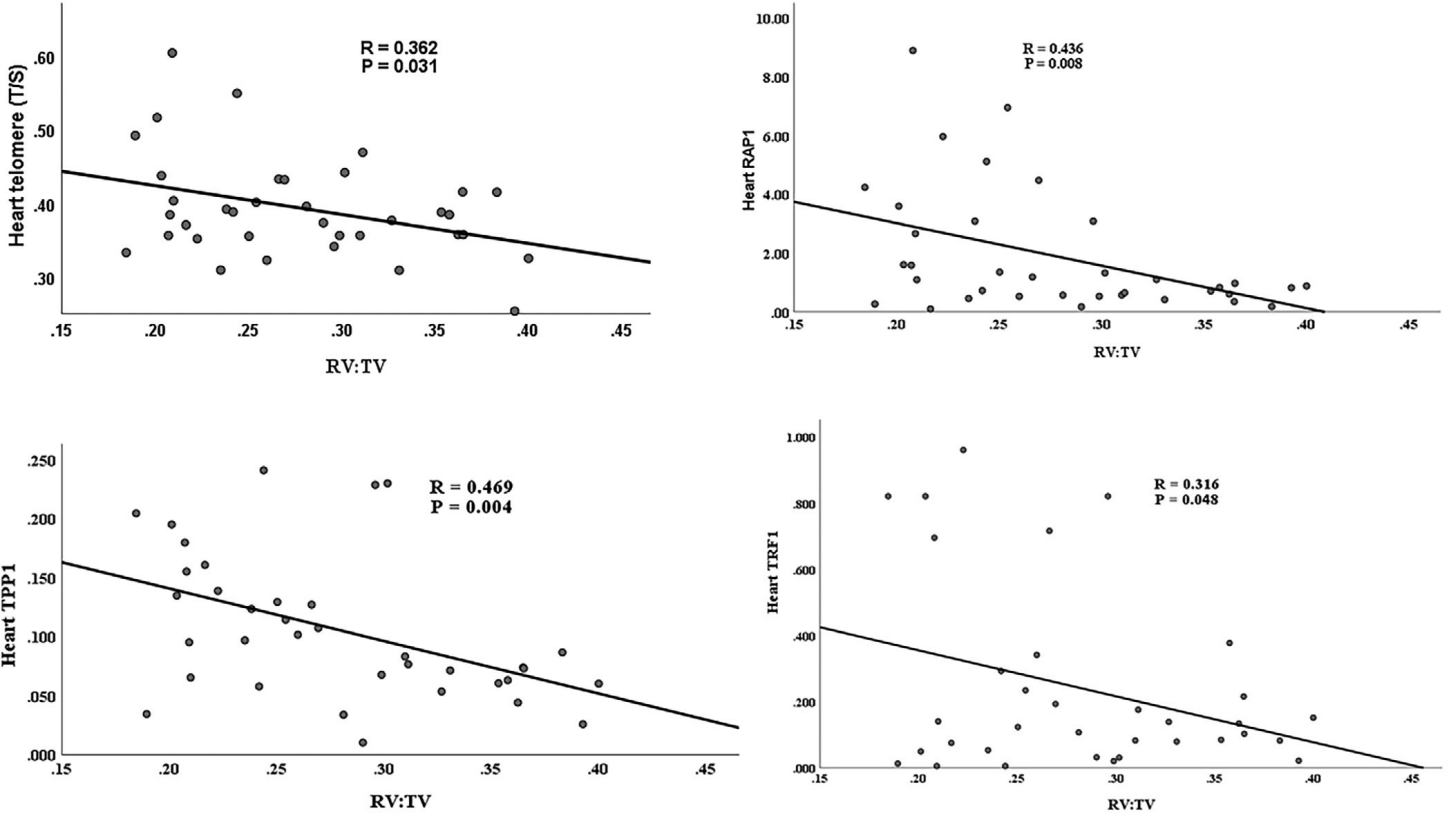
Graphs of linear regression between cardiac telomere length/relative expression of genes and RV:TV ratio (right ventricular to total ventricular weight ratio) at 42 days in the melatonin groups. *p* < 0.05 indicates a significant correlation. A negative correlation was observed between relative telomere length, TRF1, RAP1, TPP1, and the RV:TV ratio in the melatonin‐treated broilers.

Regression analysis (Figure 1) revealed a significant negative correlation between relative telomere length, *TRF1*, *RAP1*, *TPP1*, and the RV:TV ratio in the broilers treated with melatonin (*p* < 0.05). This further supports the notion that increased telomere length and shelterin gene expression are associated with reduced PHS severity.

**FIGURE 2:**
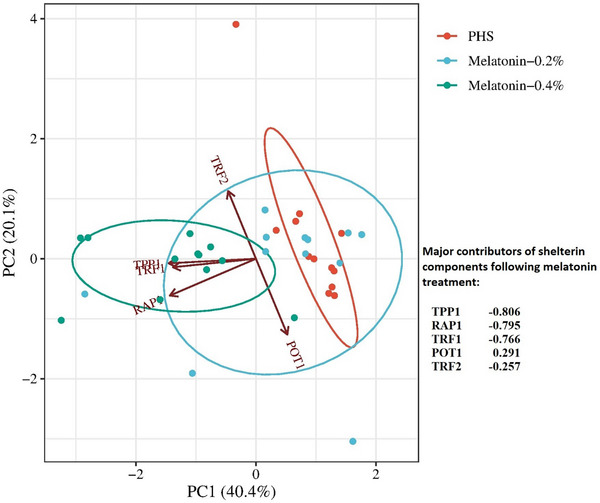
Principle component analysis (PCA) of the five shelterin components. The most contributing components to intergroup variation in pulmonary hypertensive chickens following dietary supplementation by melatonin. Ellipses represent the 68% CI of distribution in each group. The TPP1, RAP1, TRF1, POT1, and TRF2 were the top contributors to PC1, in that order.

Figure 2 shows the PCA of the five shelterin components. The most contributing components to intergroup variation were determined in pulmonary hypertensive chickens following dietary supplementation by melatonin. The TPP1, RAP1, TRF1, POT1, and TRF2 were the top contributors to PC1, in that order.

**FIGURE 3:**
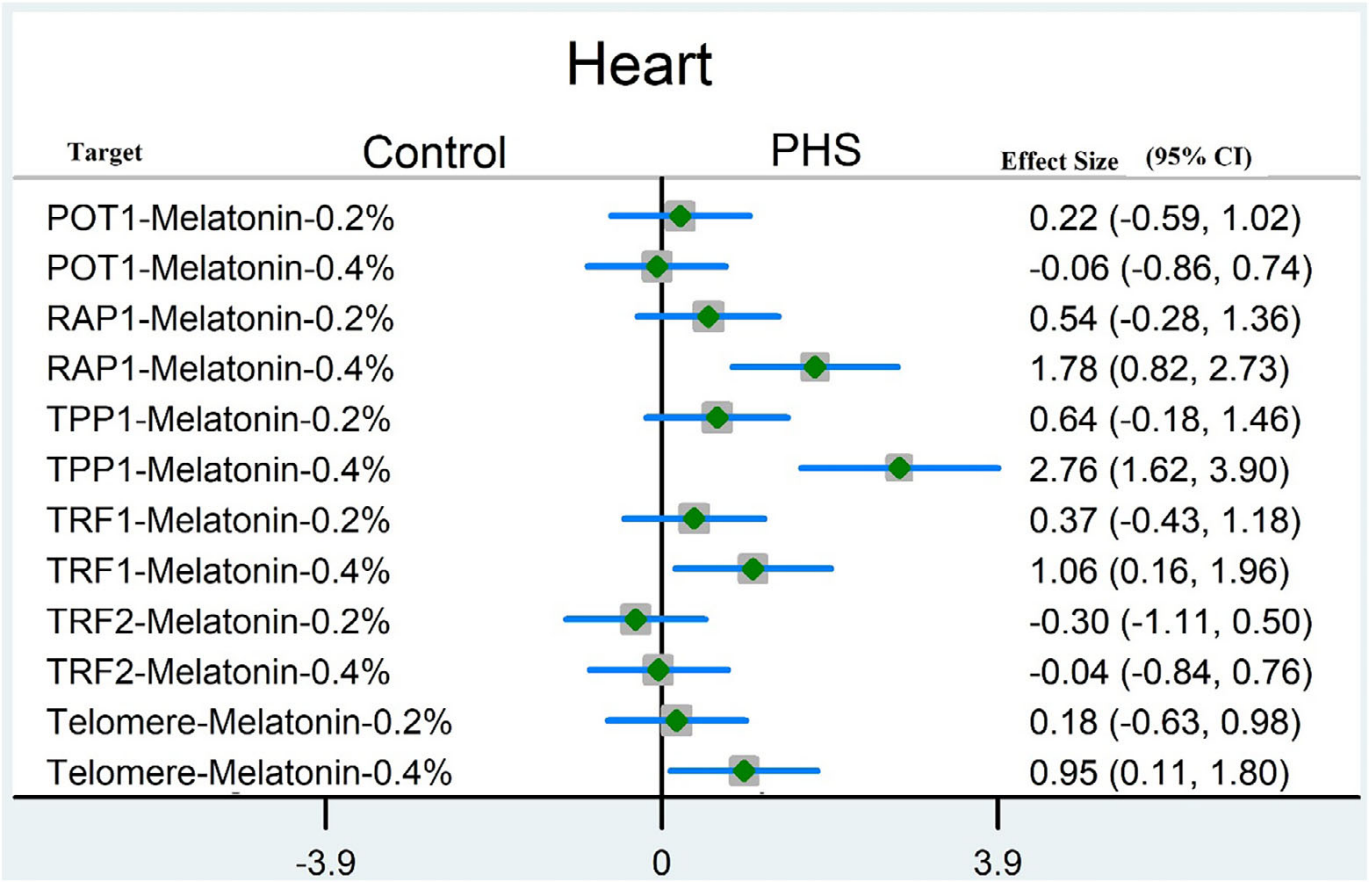
Forest plot comparing telomere length and shelterin genes in melatonin‐(0.2 and 0.4%) groups of broiler chickens. CI, coefficient interval. The effect size of telomere length, RAP1, TPP1, and TRF1 transcripts in melatonin‐0.4% significantly increased.

Figure 3 displays forest plots comparing telomere length and shelterin genes in the heart of melatonin‐(0.2 and 0.4%) groups of broiler chickens. The effect size of telomere length, RAP1, TPP1, and TRF1 transcripts only increased in the melatonin‐0.4% group.

## 4 Discussion

This research was designed to investigate telomere length and shelterin genes in the heart and lung of broiler chickens with PHS following melatonin supplementation. The most significant finding of this study is the positive impact of 0.4% melatonin supplementation on telomere length and the expression of shelterin genes in the heart tissue. This highlights the potential of melatonin to protect cardiac telomeres under the specific conditions of this induced PHS model. The application of cold stress to induce PHS initiates a critical pathophysiological cascade that encompasses increased metabolism, elevated tissue oxygen demand, heightened lung vasculature pressure, right ventricular pressure overload, right ventricular hypertrophy, and ultimately heart failure (Bahadoran et al. 2021). The RV:TV ratio is recognized as a reliable metric for detecting right ventricular hypertrophy, pulmonary hypertension (developmental PHS), and the prevalence of ascites (clinical PHS) in chickens, as previously documented (Bahadoran et al. 2023). Our research, utilizing the RV:TV ratio, demonstrated that melatonin administration can effectively modulate clinical PHS and decrease mortality rates. Previous research has demonstrated the therapeutic benefits of melatonin supplementation and its role in cardiovascular health (Dominguez‐Rodriguez et al. 2021; Veiga et al. 2021). Our findings align with these studies. Notably, plasma melatonin levels are often reduced in cases of acute and chronic heart failure in humans, with circulating melatonin proposed as a valuable biomarker for assessing the severity of ventricular remodelling (Dzida et al. 2013). Furthermore, melatonin administration has been shown to restore normal circadian blood pressure rhythms, enhance ventricular function, and mitigate hypertension in patients with congestive heart failure (Nduhirabandi and Maarman 2018). These beneficial effects are partly due to melatonin's ability to promote nitric oxide release, act as a vasodilator, inhibit α1‐adrenergic receptors, and increase cholinergic tone (Eghbal et al. 2016). Our findings align with these studies, increasing melatonin's positive impact on chicken PHS, particularly in reducing ascites‐related mortality. As discussed, oxidants are the main contributor to the pathogenesis of PHS. Several studies reported that melatonin is a scavenger of oxidants and an enhancer of the antioxidant system (Ghorbaninejad et al. 2020; Morvaridzadeh et al. 2020; Sahin et al. 2004; Segovia‐Roldan et al. 2021). However, this effect of melatonin may have a critical role in the modulation of PHS.

Yeh and Wang (2016) conducted a review highlighting the significance of telomeres and telomerase in the pathophysiology of cardiac failure, drawing from evidence in both animal models and human studies. In mice, telomere length shortening was linked to cardiomyopathy and increased apoptosis. Similarly, individuals suffering from heart failure demonstrated a significant decrease in telomere length (Aung et al. 2023). Furthermore, Hassanpour et al. (2023) supported these findings by showing that pulmonary hypertensive broilers with heart failure experienced telomere attrition, highlighting a distinct link between telomere shortening and heart failure. On the other hand, it has been reported that melatonin may be useful in the maintenance of telomere length by enhancing the expression/activity of telomerase, scavenging free radicals, and inhibiting pro‐inflammatory cytokines (Akbulut et al. 2009; Hardeland 2019). However, while other studies have explored the general cardioprotective effects of melatonin, our research uniquely focuses on its specific impact on telomere length and shelterin gene expression in the context of PHS. We also distinguished between the effects of different melatonin concentrations (0.2% vs. 0.4%), revealing a dose‐dependent response not extensively explored in previous literature.

In the previous study, significant down‐regulation of many shelterin genes, including TPP1, RAP1, and POT1, was observed in the hearts of chickens with developmental PHS (Hassanpour et al. 2023). It was reported that TPP1, a crucial telomere‐capping component involved in telomere protection, is reduced in dystrophic cardiomyopathy, and this reduction is associated with telomere attrition (Chang et al. 2016). Similarly, RAP1, another essential component of the shelterin complex, protects telomere ends by activating various DNA repair mechanisms. RAP1 also plays multiple roles in controlling gene expression, specific signalling pathways (e.g., the NF‐κB pathway), and metabolism. The NF‐κB signalling pathway appears to be a key mediator of ageing, activated by genotoxic, oxidative, and inflammatory stresses. RAP1 plays a role in reducing the activity of this pathway. Additionally, RAP1 is essential for preventing non‐homologous end joining and homologous recombination at telomeres, thereby preserving telomere integrity. However, when RAP1 is depleted, it has been demonstrated to lead to telomere shortening and subsequent cellular aging (Mir et al. 2020).

POT1, a vital component of the shelterin complex, also plays a crucial role in regulating telomere length and capping. This regulation is essential for protecting chromosome ends from recombination, instability, and abnormal segregation (Yu et al. 2017). These dysregulations of shelterin complex may contribute to the observed telomere attrition in chickens with advanced PHS, particularly those exhibiting an RV:TV ratio exceeding 0.28 at 42 days (Hassanpour et al. 2023).

It has been determined that reduced TRF1 expression can lead to telomere shortening, increased DNA damage response, and cellular senescence (Coluzzi et al. 2019). In hypertensive birds, the current study confirmed that dietary melatonin could improve shelterin genes such as TPP1, RAP1, and TRF1 evidence for telomere protection in cardiac cells. The PC analysis also determined that TPP1 could be the major contributor to telomere protection by melatonin. There are no distinct studies that explicitly explain the effects of melatonin on the shelterin complex. However, it is predictable that this hormone could benefit the gene expression of shelterin components along with providing telomere protection.

Our study found no significant changes in telomere length or shelterin gene expression in the lungs of chickens with PHS following melatonin supplementation. However, further investigation is needed to confirm these findings in other models. To date, no previous research has shown variations in these parameters in the lungs of pulmonary hypertensive chickens. Mouraret et al. (2015) reported in the lungs of pulmonary hypertensive mice that telomere length remains unchanged. Nevertheless, it is necessary to evaluate the effects of melatonin on telomere length and the expression of shelterin genes in the lungs of other animal models of pulmonary hypertension in future studies.

It is important to note that while our findings suggest a protective effect of melatonin on cardiac telomeres in this specific PHS model, these results may not be generalizable to all conditions or species.

These findings have several potential implications for poultry management practices. Supplementing broiler feed with melatonin, particularly at a concentration of 0.4%, could be a practical strategy to mitigate the incidence and severity of PHS, especially in conditions where cold stress is a factor.

This study has limitations as it focused only on broiler chickens, limiting generalizability, assessed melatonin over a short 42‐day period, leaving long‐term effects unclear, observed changes in shelterin gene expression but requires further research to clarify mechanisms, and did not measure melatonin levels, so bioavailability remains unconfirmed. However, Future research should explore the effects of melatonin on other organ systems affected by PHS, such as the liver and kidneys. Furthermore, investigating the impact of melatonin under different stress models, such as hypoxia‐induced PHS, would provide a more comprehensive understanding of its potential benefits. Exploring the effects of melatonin on different breeds of broiler chickens, particularly those more and less susceptible to PHS, could also yield valuable insights.

## 5 Conclusion

This study demonstrates the cardioprotective effect of melatonin supplementation, particularly at a concentration of 0.4%, on broiler chickens with PHS. The key contribution of this research is the evidence that melatonin improves telomere length and modulates shelterin gene expression in the heart, providing a potential therapeutic avenue for managing PHS‐related cardiac complications.

## Author Contributions

Conceptualization: Shahab Bahadoran and Hossein Hassanpour. Data curation: Peyman Gheytaspour and Shahab Bahadoran. Formal analysis: Hossein Hassanpour, Peyman Gheytaspour, and Shahab Bahadoran. Investigation: Shahab Bahadoran and Hossein Hassanpour. Methodology: Shahab Bahadoran, Hossein Hassanpour, and Peyman Gheytaspour. Supervision: Shahab Bahadoran. Writing manuscript: Hossein Hassanpour, Shahab Bahadoran, and Peyman Gheytaspour.

## Ethics Statement

This experiment was approved by the Institutional Animal Care and Use Committee of Shahrekord University (letter No. IR.SKU.REC.1400.06) by the standard of the 1964 Declaration of Helsinki.

## Conflicts of Interest

The authors declare no potential conflict of interest.

### Peer Review

The peer review history for this article is available at https://www.webofscience.com/api/gateway/wos/peer‐review/10.1002/vms3.70355.

## Supporting information



Supporting information

Supporting information

## Data Availability

Data are available upon request.
