# Supplementary material for: The Impact of Dietary Melatonin on Heart and Lung Telomere Length and Shelterin Protein Gene Expression of Pulmonary Hypertensive Broiler Chickens
Source: Vet Med Sci. 2025 Apr 21;11(3):e70355. doi: 10.1002/vms3.70355 (PMC12010761; doi:10.1002/vms3.70355)
Supplement: Supplementary file 1 — Supporting information [file VMS3-11-e70355-s001.docx]

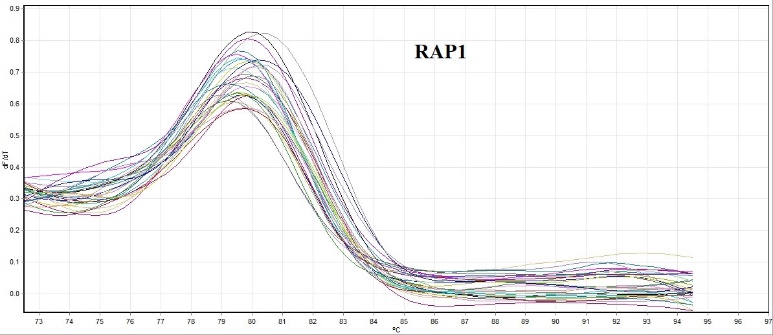

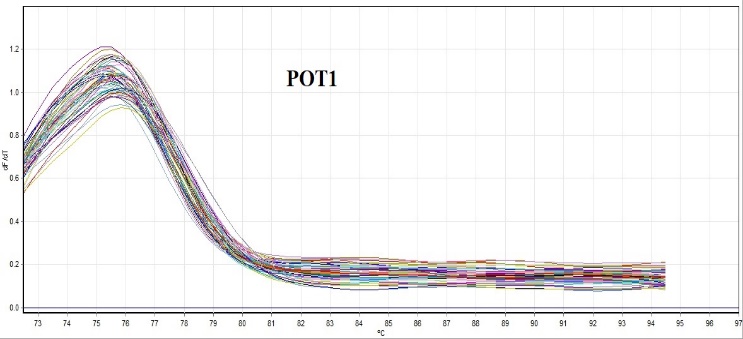


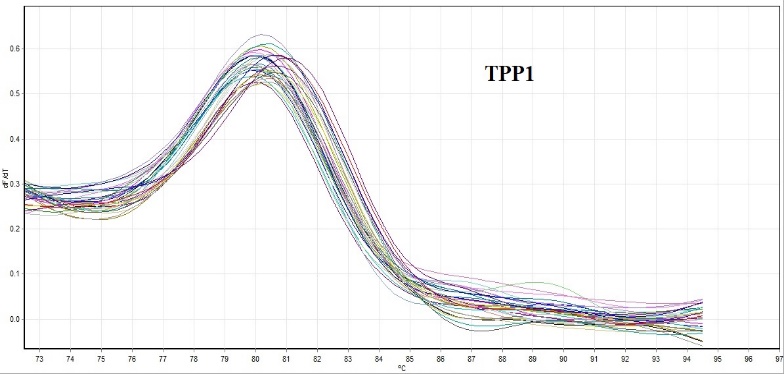

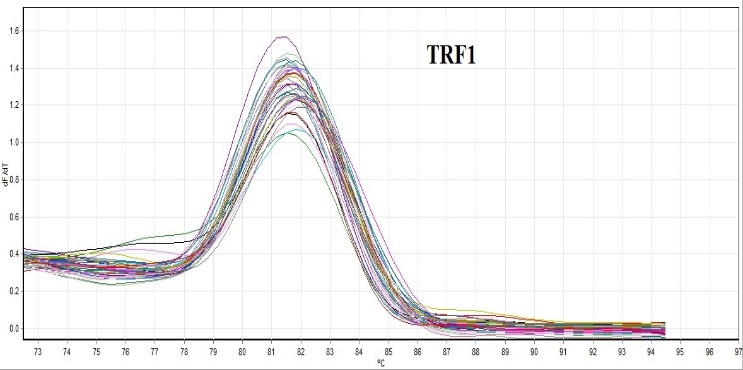


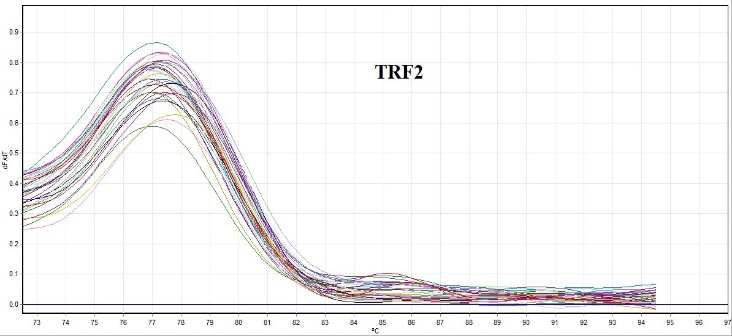

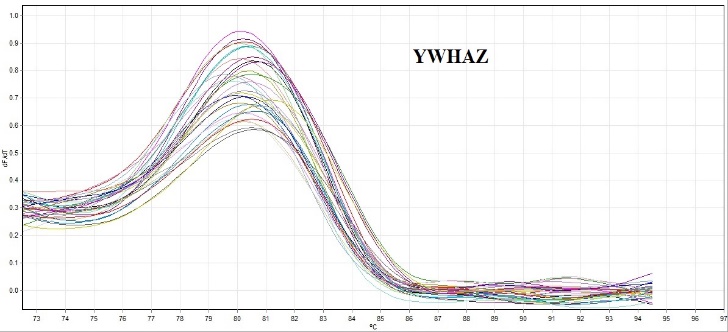


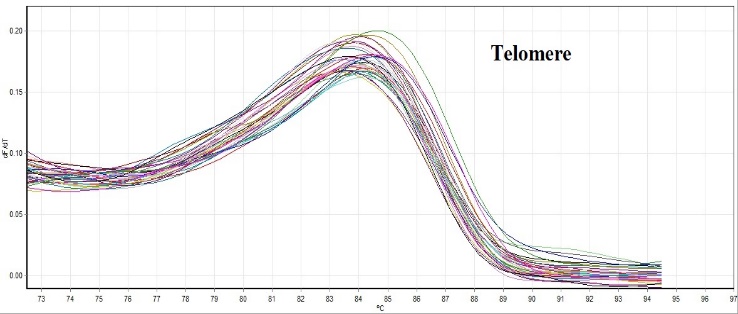


FIGURE S1 Specificity of real time PCR amplification. Melting curves (dissociation curves) of the 6 target genes and 1 reference gene (ywhaz) amplicons after the real time PCR reactions, all showing one peak. X-axis (horizontal): temperature (C); Y-axis (vertical): negative derivative of fluorescence over temperature (dF/dT)
