# Supplementary material for: The Impact of Dietary Melatonin on Heart and Lung Telomere Length and Shelterin Protein Gene Expression of Pulmonary Hypertensive Broiler Chickens
Source: Vet Med Sci. 2025 Apr 21;11(3):e70355. doi: 10.1002/vms3.70355 (PMC12010761; doi:10.1002/vms3.70355)
Supplement: Supplementary file 2 — Supporting information [file VMS3-11-e70355-s002.docx]

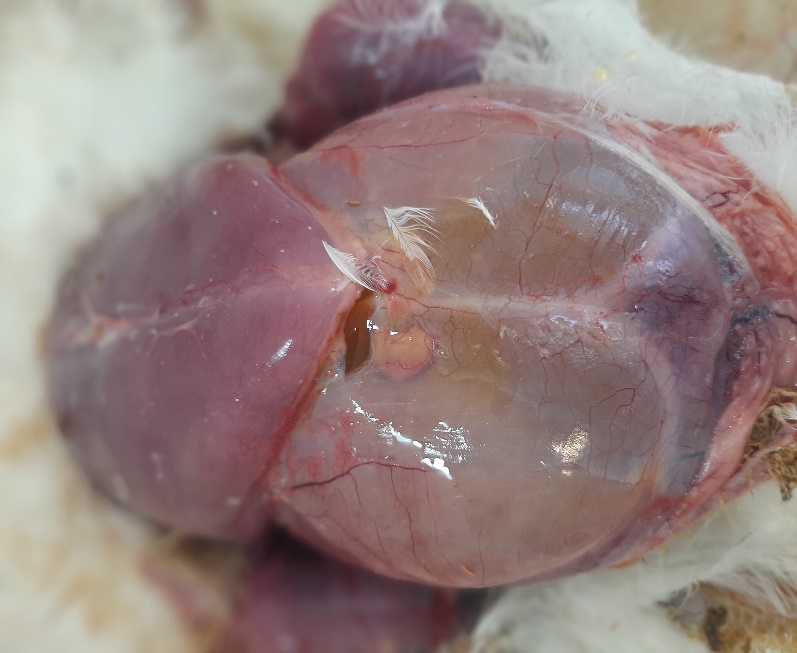

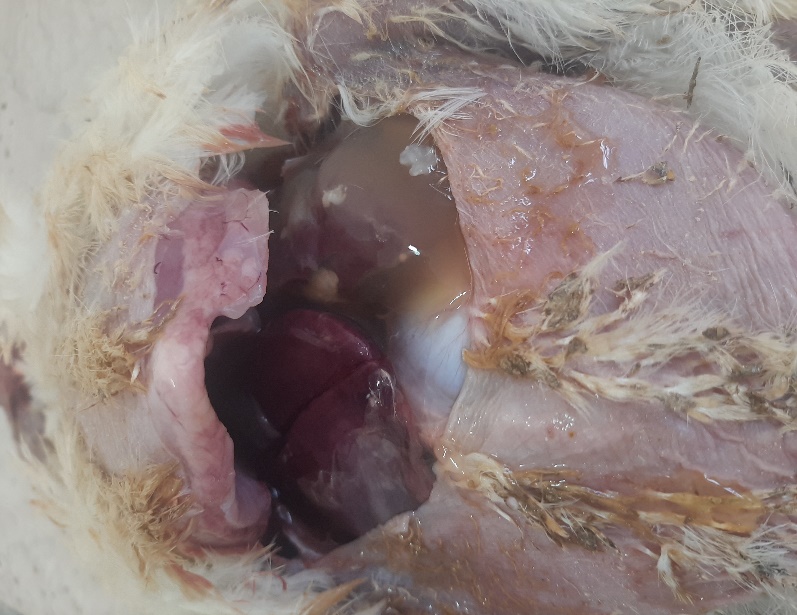

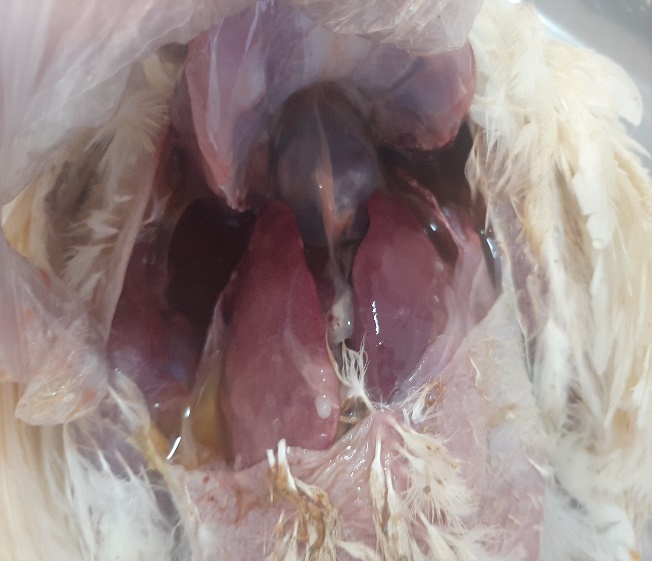

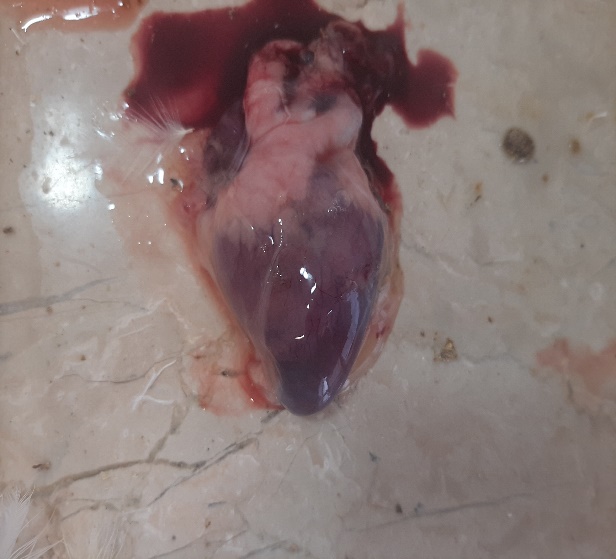


**FIGURE S2.** Different lesions of ascites in the broiler chickens under cold stress after 42 days. lesions, including fluid accumulation in the abdominal cavity, hydropericarditis, liver congestion, and enlarged heart.
